# RIPK1 and TRADD Regulate TNF-Induced Signaling and Ripoptosome Formation

**DOI:** 10.3390/ijms222212459

**Published:** 2021-11-18

**Authors:** Maria Feoktistova, Roman Makarov, Amir S. Yazdi, Diana Panayotova-Dimitrova

**Affiliations:** Department of Dermatology and Allergology, University Hospital RWTH Aachen, Pauwelsstraße 30, 52074 Aachen, Germany; mfeoktistova@ukaachen.de (M.F.); rmakarov2000@gmail.com (R.M.); ayazdi@ukaachen.de (A.S.Y.)

**Keywords:** TNF signaling, NF-κB, apoptosis, necroptosis, ripoptosome, NIK

## Abstract

TNF is a proinflammatory cytokine that is critical for the coordination of tissue homeostasis. RIPK1 and TRADD are the main participants in the transduction of TNF signaling. However, data on the cell fate-controlling functions of both molecules are quite controversial. Here, we address the functions of RIPK1 and TRADD in TNF signaling by generating RIPK1- or TRADD-deficient human cell lines. We demonstrate that RIPK1 is relevant for TNF-induced apoptosis and necroptosis in conditions with depleted IAPs. In addition, TRADD is dispensable for necroptosis but required for apoptosis. We reveal a new possible function of TRADD as a negative regulator of NIK stabilization and subsequent ripoptosome formation. Furthermore, we show that RIPK1 and TRADD do not appear to be essential for the activation of MAPK signaling. Moreover, partially repressing NF-κB activation in both RIPK1 and TRADD KO cells does not result in sensitization to TNF alone due to the absence of NIK stabilization. Importantly, we demonstrate that RIPK1 is essential for preventing TRADD from undergoing TNF-induced ubiquitination and degradation. Taken together, our findings provide further insights into the specific functions of RIPK1 and TRADD in the regulation of TNF-dependent signaling, which controls the balance between cell death and survival.

## 1. Introduction

Tumor necrosis factor (TNF) is a proinflammatory cytokine of critical importance for maintaining tissue homeostasis. [1]. Through binding to the surface receptors TNF receptor 1 (TNFR1) and TNF receptor 2 (TNFR2), TNF can initiate intracellular signaling pathways that regulate cell death and survival as well as cellular differentiation, proliferation, and immune responses. Upon activation of TNFR1, an intracellular protein complex, known as complex I, which contains receptor interacting protein 1 (RIPK1), TNFR1-associated death domain (TRADD), and other signaling molecules, is rapidly formed and activates the induction of inflammatory and survival genes. Subsequently, an assembly of different types of complexes II, namely, complex IIa, IIb (the ripoptosome), or IIc (the necrosome) [2], follows the signaling from complex I [3]. TNF co-treatment with cycloheximide (CHX) results in synthesis inhibition of a short living protein cFLIP, which is a known inhibitor of caspase-8. This leads to formation of the complex IIa and subsequent apoptosis [3]. The ripoptosome, which is formed upon the depletion of cellular inhibitors of apoptosis (cIAPs) and various extracellular stimuli [4,5], is one of the important determinators that drives the cell to either apoptosis or necroptosis.

Receptor interacting protein 1 (RIPK1) and TNFR1-associated death domain (TRADD) are essential molecules for TNF signal transduction. RIPK1 has both protein kinase and scaffolding functions. The best studied RIPK1 function is its role in the induction of necroptosis [6,7]. However, the significance of RIPK1 in TNF-dependent NF-κB signaling is still controversial. Depending on the experimental system, RIPK1 was reported to be either dispensable or unequivocally required for NF-κB activation upon TNF stimulation [8,9,10,11,12].

TRADD is an adaptor molecule that binds to activated TNFR1 and subsequently recruits further signaling molecules to the complex [13,14]. Similar to RIPK1, TRADD is a main participant in TNFR1 signal transduction and is involved in both NF-κB activation and cell death signaling [15,16]. The importance of TRADD in the regulation of TNF-induced cell signaling appeared to be cell type-dependent in both the in vitro and in vivo experimental systems and may correlate with the amount of RIPK1 in the respective cell type [12,17,18,19,20,21]. TRADD and RIPK1 were also suggested to have redundant or competing activities [12,17].

In this study, we addressed the function of RIPK1 and TRADD in TNF signaling by generating RIPK1- or TRADD-deficient human cell lines, respectively. We show that RIPK1 is relevant for TNF-induced apoptosis and necroptosis in conditions of depleted IAPs but is not absolutely necessary for the activation of NF-κB or MAPK signaling. In addition, we show that TRADD is dispensable for necroptotic cell death signaling but not for apoptotic cell death signaling. We show that similar to RIPK1, TRADD appears to not be critically required for the activation of NF-κB and MAPK signaling. Of note, partial repression of canonical NF-κB activation in both RIPK1 and TRADD KO cells does not result in sensitization to TNF alone due to the absence of NIK stabilization. Moreover, we confirm that NIK stabilization is the major prerequisite for ripoptosome formation. We reveal that RIPK1 is essential for protecting TRADD from TNF-induced ubiquitination and degradation.

## 2. Results

### 2.1. RIPK1 Promoted Both TNF-Induced Apoptosis and Necroptosis upon cIAP Depletion but Is Not Essential for NF-κB and MAPK Signaling

**Figure 1:**
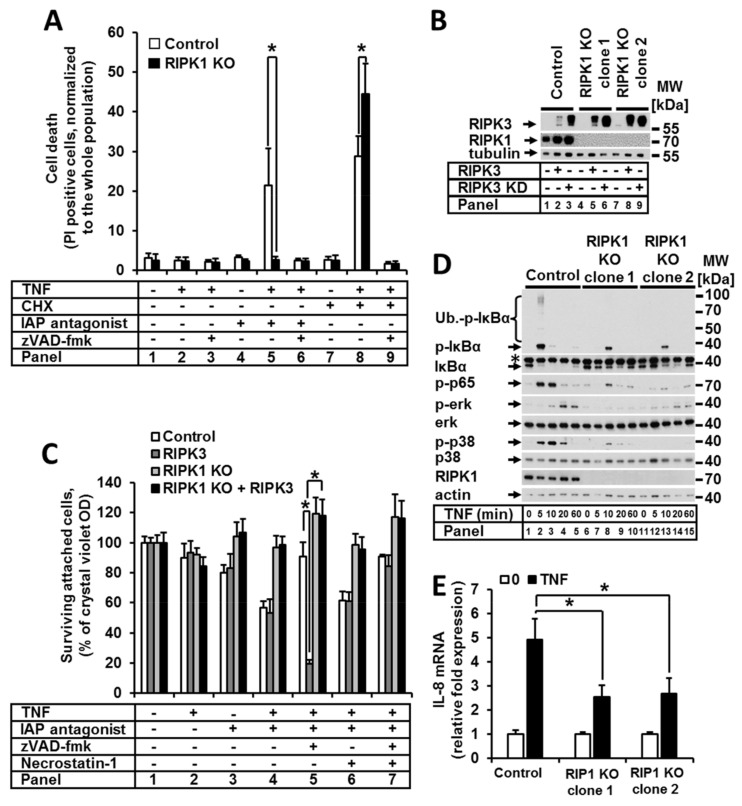
RIPK1 promoted TNF-induced apoptosis and necroptosis and non-cell death signaling. (**A**) RIPK1 KO HeLa and control cells were treated as shown, and cell death was analyzed by PI staining and FACS analysis. (**B**) Protein expression in control cells and RIPK1 KO clones overexpressing RIPK3 or RIPK3 KD was analyzed by WB. (**C**) The cells from (**B**) were treated as described and cell viability was analyzed by CV staining. (**D**) Control cells and RIPK1 KO clones were treated with TNF for the indicated time points, and cell lysates were analyzed by WB. (**E**) Control cells and RIPK1 KO clones were treated with TNF for 1 h, and mRNA expression of *CXCL8* (IL-8) was analyzed by real-time qPCR. For each diagram, the mean values (±SEM) of three independent experiments are shown. The WB shown are representative of at least two independent experiments. Error bars represent the SEM. * *p* < 0.05.

We generated HeLa cells deficient in RIPK1 using CRISPR/Cas9 technology and examined the induction of apoptosis in RIPK1 KO cells stimulated with TNF alone or in combination with the protein synthesis inhibitor cycloheximide (CHX) or IAP antagonist. In order to quantify the number of dead cells, PI staining and FACS analysis were performed (Figure 1A). As expected, in both conditions, the control HeLa cells were sensitive to TNF-induced apoptosis, which was completely blocked by zVAD-fmk (zVAD) (Figure 1A, panels 5 and 6). The absence of RIPK1 completely prevented sensitization to TNF by the IAP antagonist. However, pretreatment of RIPK1 KO cells with CHX resulted in a significant increase in sensitivity to TNF-induced apoptosis (Figure 1A, panels 7 and 8). zVAD completely blocked TNF/CHX-mediated cell death (panel 9). These data suggest that RIPK1 is a critical factor for cIAP-dependent TNF-induced apoptotic signaling; however, RIPK1 also plays a role in protecting against CHX-dependent apoptosis.

Since the IAP antagonist can induce necroptosis through RIPK3 activation, we next analyzed the effect of RIPK1 deficiency on TNF-induced necroptotic signaling. We thus expressed either a functional RIPK3 or kinase-dead (KD) RIPK3 mutant in HeLa cells, which endogenously lack RIPK3 expression (Figure 1B). We then studied quantitative and qualitative necroptosis responses to TNF stimulation. To analyze the number of surviving cells upon the respective stimulation, we used a crystal violet assay (Figure 1C). Loss of IAPs promoted TNF-induced cell death in both the control and RIPK3-expressing HeLa cells (Figure 1C, panel 4). zVAD protected the control cells from cell death but induced necroptosis in the RIPK3-expressing cells (Figure 1C, panel 5). Combined treatment with necrostatin-1 (nec-1) and zVAD completely protected the cells from cell death (panel 7). Of note, RIPK1 deficiency in necroptosis-competent HeLa cells resulted in complete rescue from necroptosis (Figure 1C, panels 5, 6).

RIPK1 has a controversial role in apoptosis and necroptosis execution, which depends on the initial conditions in the cell. RIPK1 has a pro-cell death role (both apoptotic and necroptotic) in IAP-depleted conditions, while in the case of translation blockade with CHX, RIPK1 protects against apoptosis.

Next, we aimed to analyze the impact of RIPK1 loss on non-cell death signaling. To address this question, we analyzed the induction of both noncanonical and canonical NF-κB signaling in RIPK1-deficient cells upon TNF stimulation in a time-dependent manner. We observed that loss of RIPK1 was irrelevant for the stabilization of NIK, which is a prerequisite for the noncanonical signaling pathway (Figure S1A). In contrast, the activation of canonical NF-κB signaling was significantly suppressed but not completely blocked (Figure 1D). Upon TNF treatment, both analyzed clones of RIPK1-deficient cells showed reduced IκBα phosphorylation and degradation as well as p65 phosphorylation (Figure 1D). These results were further confirmed by a significant reduction in the expression of the NF-κB target gene *CXCL8* upon TNF stimulation (Figure 1E). These data agree with the previously reported partial suppression of NF-κB signaling in HeLa-RIPK3-RIPK1 KO cells [12]. Furthermore, we analyzed the activation of MAPK signaling and observed that ERK and p38 phosphorylation/modification was partially suppressed in RIPK1-deficient cells. These observations suggest that RIPK1 plays an important but not critical role in the regulation of NF-κB and MAPK.

### 2.2. RIPK1 Is Dispensable for TNF Complex I and IIa formation but Is Critical for the Formation of a Functional Ripoptosome

**Figure 2:**
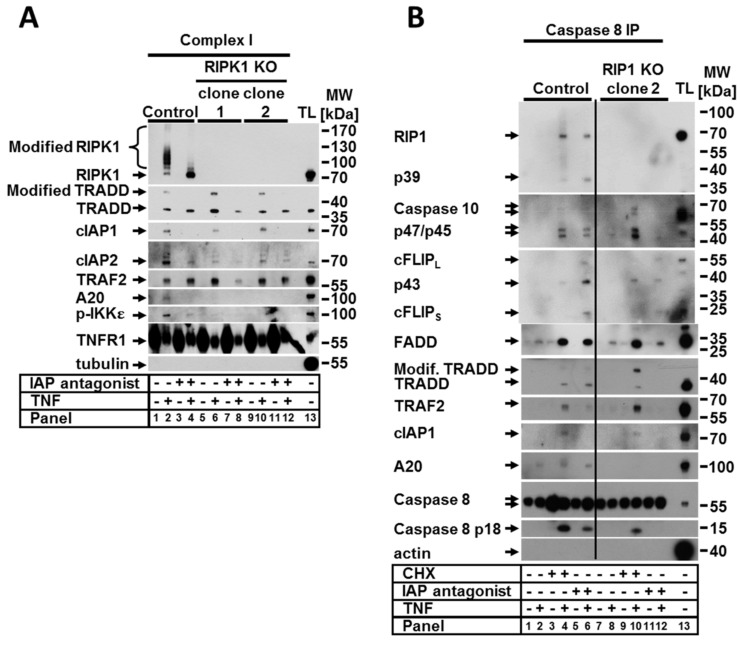
The absence of RIPK1 was not relevant for TNF complex I and IIa formation but was critical for the formation of functional ripoptosome. Control cells and RIPK1 KO clones were stimulated as indicated. (**A**) TNF complex I and (**B**) caspase-8 immunoprecipitation (IP) was performed as described in Section 4. Recruited proteins were analyzed by WB. The WB shown are representative of at least two independent experiments.

Since the activation of RIPK1-mediated signaling upon TNF stimulation is regulated in TNFR1-associated complex I, we sought to analyze how the lack of RIPK1 protein impacts the complex formation. To address this, TNF complex I was precipitated from the control and RIPK1 KO cells treated with TNF alone or in combination with the IAP antagonist. To this aim, ligand-affinity precipitation using TNF-Fc was performed. The loss of RIPK1 resulted in an altered composition of TNF complex I independent of the presence of the IAP antagonist (Figure 2A). The complexes formed upon TNF stimulation in both control and RIPK1-deficient cells contained, as expected, cIAP1, TRADD, and TRAF2 molecules. However, loss of RIPK1 repressed the recruitment of cIAP2 and completely impaired the binding of A20 and phospho-IKKε in the complex (Figure 2A). These data confirm the critical scaffolding function of RIPK1 in complex I, which is required for the recruitment of A20 and for either recruitment or phosphorylation of IKKε. Of note, the binding of cIAP2 to the complex was significantly reduced but not completely abolished, suggesting that RIPK1 may indirectly regulate cIAP2 recruitment.

RIPK1 is also known to play a critical role in the formation and activity of TNF complex IIa and complex IIb [3]. The assembly of complex IIa, which requires CHX and TNF co-treatment serves as a switch from a pro-survival response to a proapoptotic response [3,22,23,24]. Complex IIb, also known as the ripoptosome, is formed upon depletion of IAPs and/or NIK stabilization, as our group has recently reported [25], and with a combination of different conditions, such as TNF stimulation. To address the effect of RIPK1 loss on the formation of complexes IIa and IIb, we performed coimmunoprecipitation (IP) of the caspase-8 interacting molecules under various conditions (Figure 2B). Upon treatment with CHX, the composition of the assembled complex IIa was not affected by the loss of RIPK1 with the exception of A20 (Figure 2B, panels 4 and 10). The overall amount of complex IIa significantly increased in RIPK1 KO cells compared to the control cells. These data suggest that either RIPK1 is dispensable in the assembly of complex IIa upon TNF stimulation or it inhibits formation of the complex. This may explain the observed increase in apoptotic cell death in RIPK1 KO cells compared to the control upon treatment with TNF and CHX (Figure 1A, panel 8). Since RIPK1 is one of the core proteins of the ripoptosome, complex formation was largely suppressed in RIPK1-deficient cells. In the absence of RIPK1, we observed the formation of a complex containing caspase-8, cFLIP, FADD, and caspase-10, which we called a “pseudo” complex (Figure 2B, panel 12). Full-length and cleaved forms of cFLIP and caspase-10 were required for the pseudocomplex. In contrast, caspase-8 was present only in its full-length form, suggesting that its catalytic activity in the complex is suppressed when RIPK1 is absent. This observation correlates with the lack of cell death (both apoptotic and necroptotic) in RIPK1-deficient cells treated with TNF and the IAP antagonist (Figure 1A).

### 2.3. RIPK1 Protected against TRADD Modification and Degradation upon TNF Signaling

**Figure 3:**
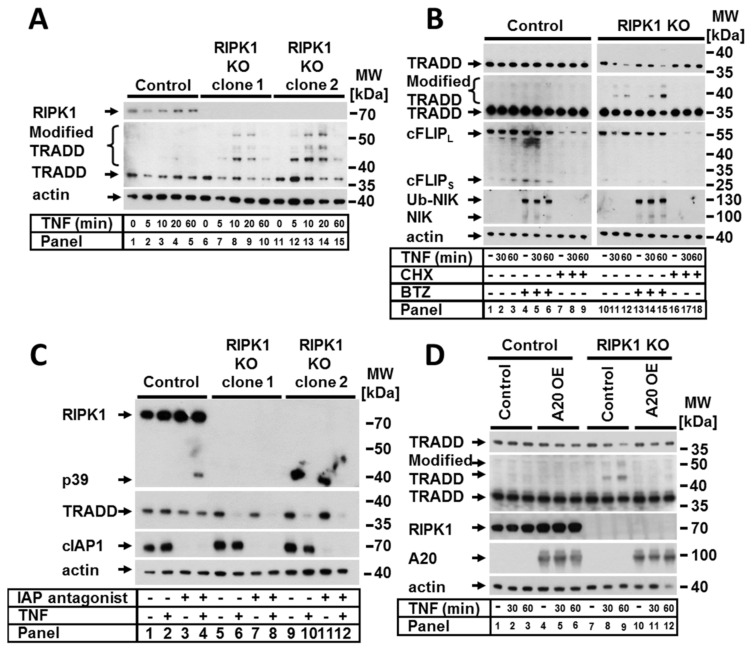
RIPK1 prevented TNF-dependent TRADD modification and degradation independent of cIAP and could be prevented by A20 over-expression. Control cells and RIPK1 KO clones were treated (**A**) with TNF for the indicated time points. (**B**) The cells from (**A**) were pretreated with BTZ and CHX for 5 h before stimulation with TNF for the indicated time points. (**C**) The cells from (**A**) were pretreated with IAP antagonist before stimulation with TNF for 2 h. (**D**) Control and RIPK1 KO cells were transduced with control or A20 containing LV and stimulated with TNF for the indicated time points. Protein expression was analyzed by WB. The WB shown are representative of at least two independent experiments.

Previous studies have suggested that crosstalk between RIPK1 and TRADD is essential for the transmission of TNF signaling [12,17,26]. We observed that in RIPK1-deficient cells, TRADD (a binding partner of RIPK1 within the complex I) was strongly and promptly modified upon TNF stimulation; this was demonstrated by the appearance of multiple fragments, suggesting polyubiquitination (Figure 3A). In both analyzed RIPK1-deficient clones, TRADD modifications appeared after 5 min of TNF stimulation and increased in a time-dependent manner for up to 20 min with a concomitant decrease in the amount of nonmodified TRADD. Interestingly, we observed that TRADD modification within complex I upon TNF stimulation was also detectible in the control cells and was significantly increased upon deletion of RIPK1 (Figure 2A, lanes 2, 6 and 10). In addition to complex I in the control cells, we detected modified TRADD within TNF complexes IIa and IIb (Figure 2B, lanes 4 and 6).

To further examine the cause of the RIPK1-dependent decrease in nonmodified TRADD upon TNF stimulation, we studied the effect of the protease inhibitor bortezomib (BTZ) and the transcription and translation inhibitor CHX (Figure 3B). Blocking proteasome function with BTZ resulted in the accumulation of modified TRADD in a time-dependent manner (Figure 3B, panels 10–16). These observations indicated that TRADD was targeted for proteasomal degradation, which was probably mediated by ubiquitination. Remarkably, CHX was able to completely abrogate RIPK1-dependent modification of TRADD (Figure 3B, panels 16–18), suggesting that RIPK1 might block one or more short-living ubiquitin ligases involved in K48 ubiquitination and proteasomal degradation of TRADD upon TNF signaling. However, within complex IIa, TRADD was modified in the presence of CHX in RIPK1 KO cells (Figure 2B, lane 10). Based on our results, cIAPs can be excluded as likely participants because TRADD modification/degradation in the presence of IAP antagonist was not abolished in cell lysates (Figure 3C). However, the absence of IAPs repressed the modification of TRADD within TNF complex I (Figure 2A, lanes 4, 8, and 12), which was independent of RIPK1 expression. A20 represents another likely candidate because of its pleiotropic functions as a ubiquitin editing enzyme, deubiquitinase (K63), and ubiquitin ligase (K48). We therefore considered a possible role of A20 based on the observed TNF-dependent TRADD modification in RIPK1-deficient cells. To address this, we overexpressed A20 in RIPK1 KO cells. Surprisingly, A20 overexpression completely prevented the RIPK1-dependent modification and degradation of TRADD upon TNF stimulation (Figure 3D). These data suggest that A20 may be indirectly involved in the removal of ubiquitin chains from TRADD. Alternatively, a spatial interaction of overexpressed A20 may prevent the binding of other molecules such as an E3 ubiquitin ligase. Since the putative E3 ubiquitin ligase that modifies TRADD is a short-living protein and this modification is a very rapid event, we aimed to check whether the expression of this E3 ubiquitin ligase is regulated by the canonical NF-κB pathway. Therefore, we generated RIPK1 KO cells with nonfunctional NF-κB by expressing an IKK2-KD construct. TNF-dependent TRADD modification in RIPK1 KO cells was unaltered in NF-κB-incompetent cells (Figure S2), suggesting that canonical NF-κB signaling is not relevant for TRADD modification.

### 2.4. TRADD Had a Role in TNF-Induced Apoptosis, NF-κB, and MAPK Signaling but Was Irrelevant for Necroptosis

**Figure 4:**
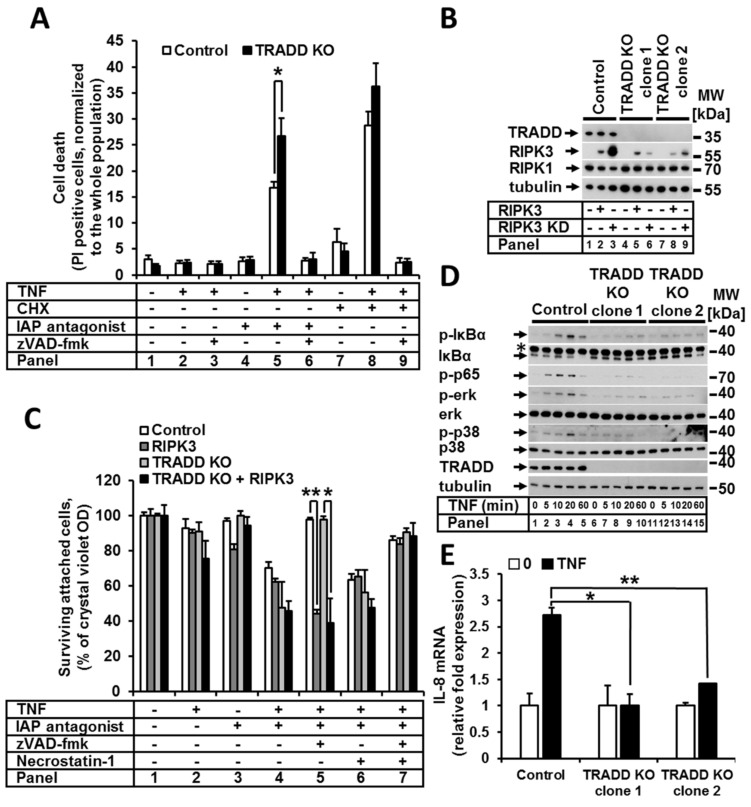
TRADD was significant for TNF-induced apoptosis and non-cell death signaling but was irrelevant for necroptosis. (**A**) Control and TRADD KO cells were treated as indicated, and cell death was analyzed by PI staining and FACS analysis. (**B**) Protein expression in control cells and TRADD KO clones overexpressing RIPK3 or RIPK3KD was analyzed by WB. (**C**) Cells from (**B**) were treated as indicated and cell viability was analyzed by CV staining. (**D**) Control and TRADD KO cells were stimulated with TNF for the indicated time points, and cell lysates were analyzed by WB. (**E**) Control cells and TRADD KO clones were treated with TNF for 1 h, and mRNA expression of *CXCL8* (IL-8) was analyzed by real-time qPCR. For each diagram, the mean values (±SEM) of three independent experiments are shown. The WB shown are representative of at least two independent experiments. Error bars represent the SEM. * *p* < 0.05, ** *p* < 0.01, *** *p* < 0.001.

To investigate the function of TRADD in TNFR1 signaling in more detail, we generated TRADD-deficient HeLa cell lines by CRISPR/Cas9 technology. We then studied quantitative and qualitative apoptotic cell death responses to TNF upon cIAP inhibition and CXH, respectively. Cell death sensitivity was analyzed by FACS analysis (Figure 4A). Loss of TRADD promoted an increase in TNF-induced apoptosis in the presence of the IAP antagonist (Figure 4A, panel 5). However, there was no significant change in apoptosis sensitivity when CHX was present (Figure 4A, panel 8). zVAD was able to protect against cell death under both CHX and IAP antagonist conditions (Figure 4A, panels 6 and 9), indicating apoptotic cell death. To address the impact of TRADD loss on TNF-induced necroptosis, we expressed functional RIPK3 and RIPK3 KD constructs in the control or TRADD-deficient HeLa cells (Figure 4B). Upon treatment with TNF alone or in combination with the IAP antagonist, RIPK3-expressing, TRADD-deficient HeLa cells showed no change in sensitivity under any of the analyzed conditions (Figure 4C), suggesting TRADD has no role in necroptotic signaling. These data collectively suggest that TRADD is involved in apoptotic but not necroptotic TNF-mediated signaling.

Next, we asked whether TNF-induced NF-κB activation is TRADD dependent. Therefore, we analyzed the impact of TRADD deletion on the activation of canonical and noncanonical NF-κB signaling. Interestingly, TRADD KO promoted NIK stabilization, which is induced by the IAP antagonist. Furthermore, IAP antagonist-induced NIK stabilization increased in TRADD KO cells compared to the control cells (Figure S1B). We observed that IκBα phosphorylation and degradation as well as p65 phosphorylation were strongly suppressed but not completely abrogated in TRADD-deficient cells (Figure 4D). Moreover, the mRNA expression of the NF-κB target gene *CXCL8* upon TNF stimulation was largely reduced in TRADD KO cells (Figure 4E). Moreover, we analyzed the activation of MAPK signaling and observed partial suppression of ERK and p38 phosphorylation/activation in TRADD-deficient cells (Figure 4D).

**Figure 5:**
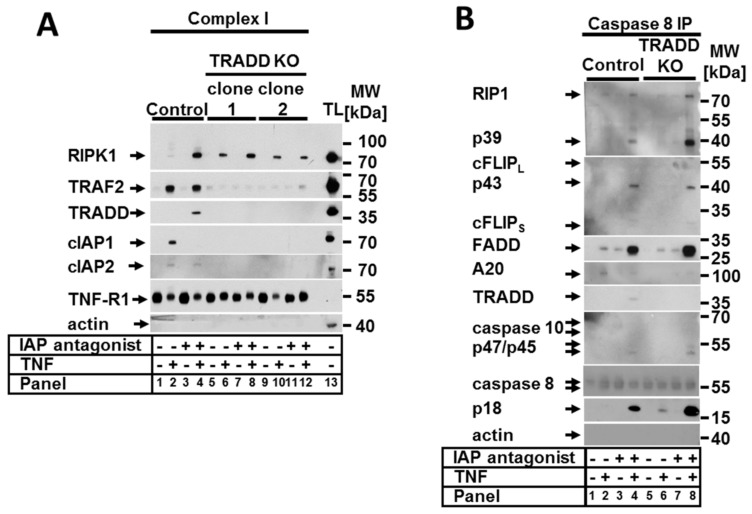
TRADD was critical for complex I formation but irrelevant for ripoptosome formation. Control and TRADD KO cells were stimulated as indicated. (**A**) TNF complex I and (**B**) caspase-8 immunoprecipitation (IP) were performed as described in Section 4. Recruited proteins were analyzed by WB. The WB shown are representative of at least two independent experiments.

Next, we investigated the composition of TNF complex I in cells lacking TRADD. As expected, without TRADD, no other molecules were recruited to TNF-R1 except for unmodified RIPK1 (Figure 5A). However, immunoprecipitation of the ripoptosome demonstrated that the loss of TRADD and the lack of TNF complex I formation enhanced rather than prevented the formation of the ripoptosome (Figure 5B). This observation explains the detected increase in the sensitivity of TRADD-deficient cells to TNF in the presence of the IAP antagonist. Moreover, these results confirm our previous data demonstrating that the formation of ripoptosome is an independent event and that the components of the ripoptosome complex are assembled autonomously and do not originate from TNF complex I [4,25].

## 3. Discussion

RIPK1 and TRADD together with FADD and TRAF2 belong to the main participants in TNFR1 signal transduction. We examined the roles of RIPK1 and TRADD in the regulation of both apoptosis and necroptosis in the presence of CHX and IAP antagonist. We found that RIPK1 was significant in the negative regulation of apoptotic cell death in the presence of CHX in human cells. These data confirm previously reported studies showing that RIPK1-deficient MEFs had increased sensitivity to TNF/CHX-induced cell death [17,24,27]. Moreover, we showed here that RIPK1 has a critical role for both apoptosis and necroptosis regulation in the absence of IAPs. In addition, we provided biochemical evidence that TNF complex IIa in RIPK1-deficient cells formed in the presence of CHX and was fully assembled. In contrast, the formation of the IIb complex (ripoptosome) in the presence of the IAP antagonist was strongly compromised and resulted in the assembly of a so-called pseudocomplex, which could not direct the cell to apoptotic cell death. Of note, because only full-sized but not cleaved caspase-8 was present in the pseudocomplex, RIPK1 may have a role in the activation of caspase-8 in the ripoptosome. Additionally, we demonstrated that TRADD-deficient cells were marginally more sensitive to TNF-dependent apoptosis in the presence of CHX, and in contrast to RIPK1, this increase in sensitivity was statistically irrelevant. Füllsack et al. recently demonstrated that the combined TNF and CHX treatment had a similar effect on necroptosis-competent HeLa cells [12]. We found here that in the presence of the IAP antagonist, TRADD KO cells demonstrated increased sensitivity to apoptotic but not necroptotic cell death. In support of these data, we observed the formation of fully assembled TNF complex IIb (ripoptosome), suggesting that TRADD repressed the apoptotic function of the ripoptosome. Of note, the fact that no TNF complex I was detected in TRADD KO cells (Figure 5A) confirms our previous observations suggesting that TNF complex IIb/ripoptosome is formed independently from TNF complex I [4,25].

It was recently reported that the loss-of-function mutations of RIPK1 in humans cause impaired innate and adaptive immunity and predisposition to immune and intestinal dysfunctions. RIPK1 KO cells from RIPK1-deficient patients demonstrated compromised NF-κB and MAPK signaling and dysregulated cytokine production [28,29]. Here, we showed that both RIPK1 and TRADD deficiencies resulted in strongly impaired TNF-induced NF-κB and MAPK activation and significantly decreased *CXCL8* expression. Under normal conditions, NEMO/IKK complex is recruited to complex I via K63 ubiquitin chains, which are linked to RIPK1 by cIAPs. TRADD is required for the recruitment of cIAPs to TNF complex I. It is tempting to speculate that complex I in the absence of RIPK1 is still able to recruit the NEMO/IKK complex, through some other modified component of TNF complex I. This would explain the presence of decreased but not abolished NF-κB signaling in RIPK1 KO cells. Of note, since we detected that complex I was composed of only nonmodified RIPK1 in TRADD-deficient cells, the presence of NF-κB signaling in those cells cannot be explained with the current model. Possibly other components are recruited to the complex but cannot be detected via the immunoprecipitation method. Further analysis for the identification of new, putative components is needed.

Of note, partial repression of canonical NF-κB activation in both RIPK1 and TRADD KO cells does not result in sensitization to TNF alone. This is in contrast to our previous observations, which demonstrated that when NF-κB was completely blocked by IKK2-KD overexpression [30] or partially blocked by A20 overexpression [25], the cells became sensitive to TNF-induced cell death by TNF alone. In both cases, we demonstrated that NIK stabilization was a consequence of increased IKK2-KD (Figure S1C) or A20 expression [25] and represented a prerequisite for ripoptosome formation upon TNF stimulation. We observed that deleting RIPK1 and TRADD did not induce NIK stabilization (Figure S1A,B) and consequently did not result in increased sensitization to TNF-induced cell death. These data further confirm our previous hypothesis that NIK stabilization is one of the critical checkpoints in TNF-induced cell death mediated by the ripoptosome. Although the deletion of TRADD did not induce NIK stabilization, it enhanced NIK stabilization caused by the absence of cIAPs (Figure S1B), which resulted in increased ripoptosome formation and cell death. Thus, it can be speculated that TRADD represents a blocker of NIK stabilization and the subsequent ripoptosome formation.

The complex crosstalk between RIPK1 and TRADD in TNF signaling has been discussed in a number of reports over the years. The ratio between both proteins in the respective cellular system appears to present a plausible explanation for the different relevance of RIPK1 or TRADD in various analyzed systems [26]. We found here that the RIPK1 expression level was relevant for TRADD modification. A RIPK1-dependent increase in TRADD modification was reported previously in Jurkat cells with downregulated RIPK1 [17]. Importantly, we demonstrated that RIPK1 was able to control TRADD protein stability upon the induction of TNF signaling. We revealed that RIPK1-dependent TNF-induced TRADD polyubiquitination appeared very rapidly, directing TRADD to proteasomal degradation. Additionally, we observed TRADD ubiquitination in TNF complexes I, IIa, and IIb in control cells expressing RIPK1. These modifications within the complexes did not appear to result in TRADD degradation, which suggests that they might belong to K63 ubiquitination events. We showed here that TRADD ubiquitination in complex I was driven by cIAPs and that the absence of RIPK1 promoted this modification.

Our data suggest that upon TNF stimulation, RIPK1 might be responsible for deactivating a short-living E3 ubiquitin ligase. When RIPK1 is missing, the ubiquitin ligase can ubiquitinate TRADD and direct it to proteasomal degradation. Since cIAPs repressed TRADD ubiquitination in complex I but did not prevent its degradation in the total lysates, we excluded the cIAPs from the E3 ubiquitin ligases that are suspected to be involved in this process. We next hypothesized that A20 might play a role in TRADD polyubiquitination. Surprisingly, the elevated expression of A20, which is known to ligate K48 chains to different proteins, completely prevented RIPK1-dependent TRADD ubiquitination and degradation. This observation suggests that the direct involvement of A20 in the RIPK1-dependent modification of TRADD can be excluded. However, it can still be assumed that A20 has a potential indirect involvement. One putative function of A20 could be the deubiquitination of another E3 ubiquitin ligase required for TRADD ubiquitination. Moreover, it is tempting to speculate that this does not occur within complexes I or II but rather in the cytoplasm, since A20 cannot be recruited to the RIPK1-deficient complex. Alternatively, a direct spatial interaction from the overexpressed A20 protein might directly prevent the binding of other E3 ubiquitin ligases, thus avoiding the modification of TRADD. In summary, we speculate that RIPK1 is able to control the ubiquitination and degradation of TRADD via yet unknown molecules. Similar involvement of the ubiquitin–proteasome pathway in the regulation of protein stability in cell death signaling has been reported previously. For example, it was shown that MKRN1 E3 ligase is involved in the ubiquitination and degradation of FADD and can influence the rate of TRAIL-dependent apoptosis in breast cancer cells [31].

**Figure 6:**
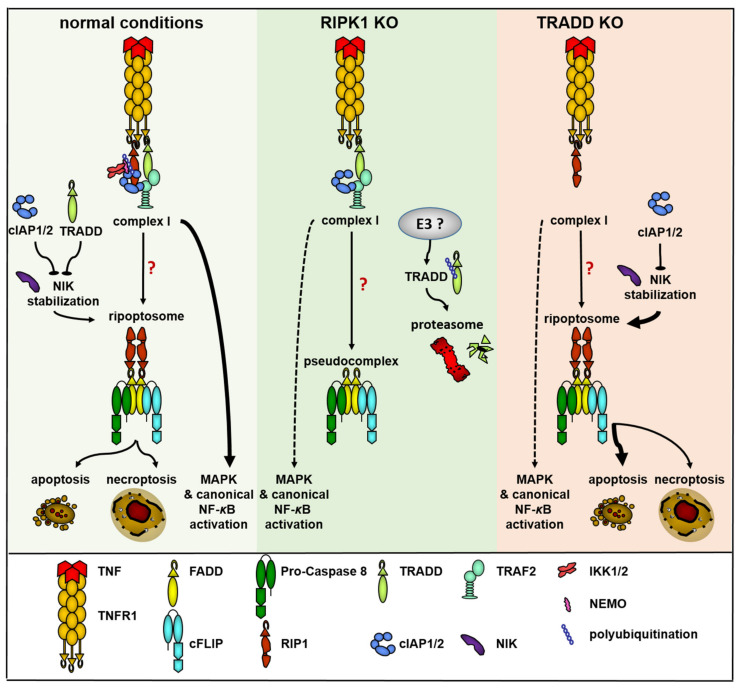
Suggested model of TRADD and RIPK1 functions in TNF signaling and ripoptosome formation. Under normal conditions (yellow field), the signal initiated by TNF through TNF-R1 proceeds via the assembly of TNF complex I and initiates NF-κB and MAPK signaling. Assembly of complex I and stabilization of NIK are the requirements for ripoptosome formation, which can direct the cell to apoptosis or necroptosis. NIK stabilization can be blocked by cIAPs or TRADD. When RIPK1 is missing (green field) complex I is assembled but NF-κB and MAPK signaling are partially blocked. No ripoptosome is formed. The assembly of pseudocomplex is unable to direct the cell-to-cell death. Unknown E3 ubiquitin ligase can ubiquitinate TRADD and direct it to proteasomal degradation. When TRADD is missing (red field), complex I consists only of unmodified RIPK1 and both NF-κB and MAPK signaling are partially blocked. NIK stabilization is simplified and ripoptosome is assembled and can direct the cell to apoptosis or necroptosis.

In summary, RIPK1 and TRADD are involved in the control of TNF signaling by interfering at multiple levels, such as the NF-κB and MAPK pathways, ripoptosome formation (NIK stabilization), and finally RIPK1, which is relevant to the stability of TRADD protein (Figure 6). Detailed investigation of the interplay between TRADD and RIPK1 in the TNF signaling pathway is needed to better understand the complex relationship resulting in the functional role of both molecules in the control of cell fate.

## 4. Materials and Methods

The following antibodies (Abs) were used for WB: RIPK1 (R41220), TRADD (T50320), and FADD (F36620) Abs were purchased from BD Transduction Laboratories, San Diego, California. Caspase-8 (C-15; kindly provided by P.H. Krammer); caspase-10 (M059-3) from MBL, Woburn, MA, USA. RIPK3 (IMG-5846A) from Imgenex, San Diego, CA, USA. Rat Abs were used against cIAP1 [32] and cIAP2 [33]. β-actin (A 2103) and β-tubulin (T4026) were from Sigma, St. Louis, USA; TRAF2 (ab126758) from Abcam, Cambridge, UK. IκBα (sc-371), caspase-8 (C20) and TNFR1 (SC-8436) were purchased from Santa Cruz, CA, USA. pIκBα (#9246), p-p65 (#3031), p100/p52 (#4882), IKK2 (#2684), and NIK (#4994) from Cell Signaling, Danvers, MA, USA. HRP-conjugated goat anti-rabbit, goat anti-rat IgG, goat anti-mouse IgG Abs, and HRP-conjugated goat anti-mouse IgG1, IgG2a, and IgG2b Abs were purchased from Southern Biotechnology Associates, Birmingham, AL, USA. The pan-caspase inhibitor Z-Val-Ala-DL-Asp-fluoromethylketone (zVAD-fmk) was purchased from Bachem GmbH, Bachem, Germany. Necrostatin-1 from Sigma, St. Louis, MO, USA. IAP antagonist (compound A) was kindly provided by TetraLogics Pharmaceuticals (Philadelphia, PA, USA). The construct for Fc-TNF expression was provided by P. Schneider [34] (University of Lausanne, Epalinges, Switzerland). HF-TNF was produced and purified as previously described [30].

### 4.1. Generation of Cell Lines

CRISPR cell line generation:

TRADD KO cells were generated using the pSpCas9(BB)-2A-GFP (PX458) plasmid (Addgene. gRNA sequences targeting the 5-end of the gene were designed using the open access software provided at http://crispr.mit.edu/ accessed date 1 March 2017. The gRNA sequences used were as follows:

TR1: GGTGCGCGTAGGCATCCGAC;

TR2: GCAAAATGGGCACGAAGAGT.

Forty-eight h after transfection, the cells were sorted with a BD FACSAria I (BD Biosciences), and single clones were isolated and analyzed to confirm successful deletion of TRADD.

The RIPK1 KO cell line was generated exactly as described in [35].

HeLa cells were provided by Michael Boutros (DKFZ, Heidelberg) and were cultured in RPMI medium containing 10% fetal calf serum (FCS).

The generation of A20- and IKK2-KD OE cell lines is explained in detail elsewhere [25]. The ectopic expression of the respective molecules was confirmed by WB. Cells from passages 2–6 were used for subsequent analyses.

### 4.2. Conditions for Cell Stimulation

The following stimulation conditions were used: pre-stimulation with zVAD-fmk (10 mM), necrostatin-1 (50 mM), and IAP antagonist (100 nM) alone or in the respective combinations for 1 h. Pre-stimulation with CHX (5 µg/mL) and BTZ (1 µM) was performed for 5 h. HF-TNF stimulation concentration for the crystal violet assay, propidium iodide (PI) staining, and WB was 250 ng/mL. Stimulation for complex immunoprecipitation was performed as follows: caspase-8 co-immunoprecipitation-1 mg/mL HF-TNF for 2 h and ligand affinity precipitation-TNF-Fc supernatant for 5 min.

### 4.3. Western Blot Analysis

For analysis of proteins, the following lysis buffer was used: 30 mM TRIS-HCL (pH 7.5), 120 mM NaCl, 10% glycerol, 1% Triton X, 2 tablets Complete (Protease Inhibitor) per 100 mL. The lysis was performed for 20 min on ice, followed by 10 min centrifugation at 14,000× *g* rpm.

For analysis of phospho-proteins, the respective cells were starved in serum free medium for 6 h. The scraped cells were resuspended in the following triton lysis buffer: 20 mm Tris (pH 7.4), 137 mm NaCl, 10% (*v*/*v*) glycerol, 1% (*v*/*v*) Triton X-100, 2 mm EDTA, 50 mm sodium β-glycerophosphate, 20 mm sodium pyrophosphate, 1 mm Pefabloc, 5 mg/mL of aprotinine, 5 mg/mL of leupeptin, 5 mm benzamidine, and 1 mm sodium orthovanadate. All lysates were homogenized by a syringe and 0.4 mm needle, followed by 10 min centrifugation at 14,000× *g* rpm.

Five micrograms of total cellular protein were separated by SDS-PAGE on 4–12% gradient gels (Invitrogen, Karlsruhe, Germany) and transferred to nitrocellulose or PVDF membranes. After the blocking step, the membranes were incubated with primary and appropriate secondary Abs as described previously [36]. Bands were visualized with an ECL detection kit (Amersham, Freiburg, Germany).

### 4.4. Complexes Precipitations

IP of caspase-8-bound complexes and TNF-Fc ligand affinity precipitation were performed exactly as explained in [25].

### 4.5. Crystal Violet Assay

Crystal violet staining of attached live cells was performed 18–24 h after stimulation with HF-TNF as described in the “cell stimulation conditions”. All experiments were completed in 96-well plates in triplicate as previously described [36]. The optical density (OD) of the control wells was normalized to 100% and used as a reference for all stimulation conditions.

### 4.6. Propidium Iodide Staining

A total of 2 × 104 cells were stimulated as described in the “cell stimulation conditions” for 18 h. After trypsinization, the cells were washed with PBS and stained with 10 µg/mL PI for 15 min (dark). BD Accuri C6 flow cytometer was used for analysis.

### 4.7. RNA Isolation and Real Time qPCR

RNA isolation from HeLa cells was performed with RNeasy Kit (Qiagen, Hilden Germany). For cDNA synthesis: SuperScript II Reverse Transcriptase (Invitrogen, Waltham, MA, USA) and a mixture of random nanomers and oligo dT primers in a ratio 10:1 was used. RT qPCR analysis was performed using PowerUp™ SYBR™ Green Mastermix (Thermo Fisher Scientific, Waltham, MA, USA) in the QuantStudio 1 Real-Time-PCR System (Thermo Fischer Scientific). Equal cycling conditions were used to amplify genes of interest and reference gene products. Mean values were calculated using data obtained from three independent experiments. Normalization of each experiment was performed to β-actin expression. Primer sequences for *CXCL8*: For-CACCCCAAATTTATCAAAGA and Rev-ACTGGCATCTTCACTGATTC: and actin: For-CGCCTTTGCCGATCC and Rev-ACGATGGAGGGGAAGAC.

### 4.8. Statistics

All data are expressed as the mean ± SEM. A two-tailed Student’s *t*-test for two groups was used to assess the significance of differences.

## Data Availability

The data are available on request.

## Supplementary Materials

The following are available online at https://www.mdpi.com/article/10.3390/ijms222212459/s1.
