# Supplementary material for: RIPK1 and TRADD Regulate TNF-Induced Signaling and Ripoptosome Formation
Source: Int J Mol Sci. 2021 Nov 18;22(22):12459. doi: 10.3390/ijms222212459 (PMC8617695; doi:10.3390/ijms222212459)
Supplement: Supplementary file 1 [file ijms-22-12459-s001.zip › ijms-1462381-supplementary.pdf]

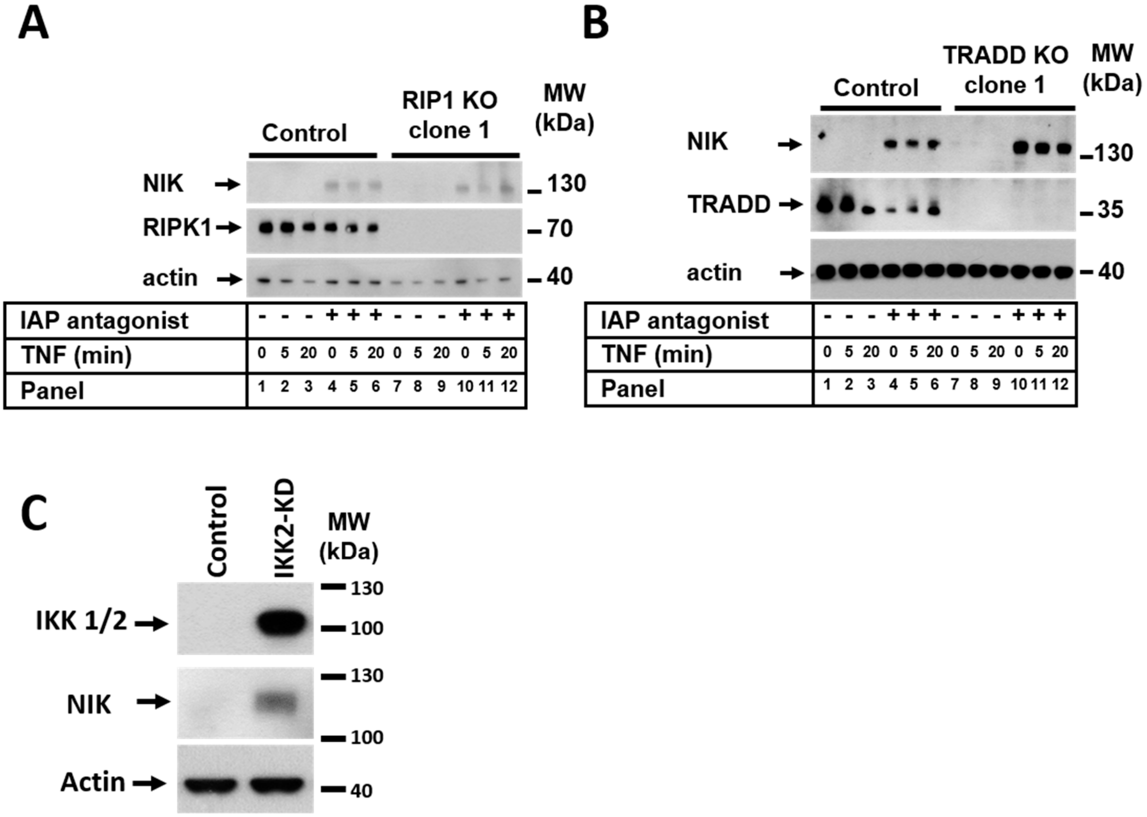

Figure S1. NIK stabilization. Control and RIPK1KO cells (**A**), TRADD KO cells (**B**) or IKK2KD (**C**) cells were treated as indicated and protein expression was analyzed by WB.

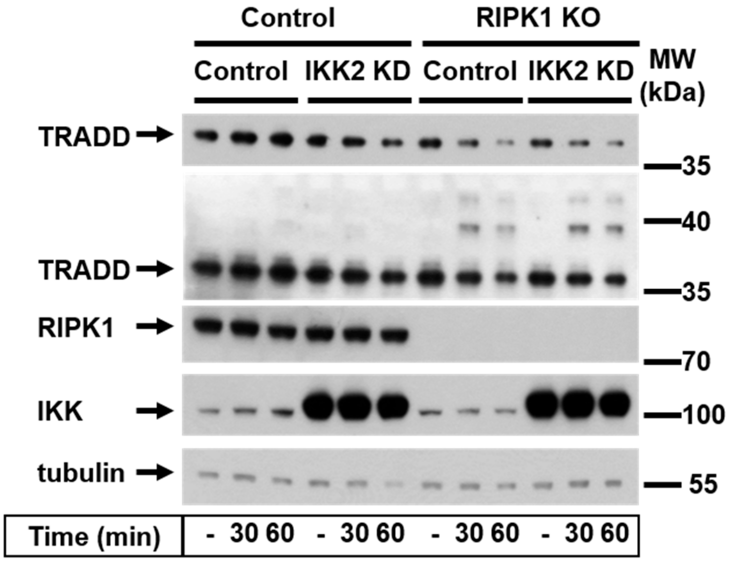

Figure S2. Canonical NF-κB signaling is irrelevant for TRADD modification. Control and RIPK1KO cells were transduced with IKK2KD or control vector and treated as indicated. Protein expression was analyzed by WB.
